# A novel “three-port” trocar placement technique for laparoscopic radical prostatectomy

**DOI:** 10.1186/s12957-020-02051-y

**Published:** 2020-10-27

**Authors:** Ben Xu, Yi-ji Peng, Guo-zhong Ma, Qian Zhang

**Affiliations:** 1 Department of Urology, National Urological Cancer Center, Peking University First Hospital and Institute of Urology, Peking University, 8 Xishiku Street, Xicheng District, Beijing, 100034 China; 2 Department of Urology, Affiliated Hospital of Heze Medical College, 777 Zhujiang Road, Mudan District, Heze City, 274000 Shandong China

**Keywords:** Three-port, Trocar, Laparoscopic, Radical prostatectomy

## Abstract

**Background:**

To introduce a novel “three-port” trocar placement technique for laparoscopic radical prostatectomy (LRP) in prostate cancer (PCa) patients.

**Methods:**

We retrospectively reviewed 300 patients with PCa who received surgical treatment between November 2010 and June 2015 at our institution. They were divided into group A, three-port LRP; group B, conventional four-five-port LRP; group C, open RP (ORP); and group D, robotic-assisted RP (RARP). A learning curve was analyzed by dividing patients of group A into the early and late stages.

**Results:**

All groups were comparable with regard to the preoperative characteristics except for the relatively smaller prostate volume in group A. The three-port LRP operations were performed successfully with only 8 cases of conversion to the conventional LRP. None of any severe complications or conversion to ORP occurred. In group A, the mean operative time (OT) duration was 113.8 min, the mean estimated blood loss (EBL) was 94.2 ml, the mean drainage days was 4.0 days, the mean hospitalization was 5.1 days, and 27.8% of the prostate specimen margins (PSM) were positive. The differences of OT, EBL, drainage days, hospitalization, and transfusion in group A were statistically significant among the majority of the other groups (*p* < 0.05). After undergoing the early stages of a learning curve analysis in three-port LRP, the EBL was obviously decreased.

**Conclusions:**

Three-port LRP is a novel technique that exhibits superior intraoperative advantages to the conventional LRP. Due to its less OT, EBL, drainage days, hospitalization, and transfusion with a shorter learning curve, it should be recommended and popularized in the clinical practice.

## Background

Prostate cancer (PCa) is the second most common malignant tumor in men and an important cause of cancer-related disease among the patients worldwide [1], accounting for approximately 12% of newly diagnosed cancer cases in men [2]. In the developed country of the USA, PCa comprises nearly 21% of newly diagnosed tumors in male patients [3]. The incidence of PCa in China is much lower than that in western countries but has increased dramatically in recent years [4]. Satisfactory therapeutic effects for PCa at early stage can be achieved through RP, including ORP, LRP, and RARP [5, 6].

RARPs were initially performed in the USA and Germany in 2000 [7], and then, it has gradually surpassed the traditional ORP and been applied extensively as a first-line treatment in several high-volume centers with improved perioperative outcomes without compromising cancer control. Although a high percentage of PCa patients have been treated with RARP, the costs are still too high. This challenge is what many urologic surgeons must face in relatively poor and underdeveloped countries. As a matter of fact, patients undergoing robotic surgery often report dissatisfaction and regret after the operation [8]. Mukherjee et al. [9] and Preisser et al. [10] have also warned that the high total cost of RARP must be kept in mind even though in the USA.

In China, the introduction of robotic systems appears to be sparse compared with that in western countries. The conventional LRP still plays an important role in China, which has the majority of PCa patients among different countries. The situation is also nearly the same in other developing countries [11]. Therefore, the conventional four-five-port LRP might likely continue to exist for many years to come due to its cost. However, the unfamiliar coordination of the surgeon and assistant with a more trocar-associated negative impact on cosmesis both urges us to improve this traditional technique. The three-port LRP is simply a modified technique that we improved, which exhibits superior perioperative advantages when compared with other minimally invasive options. In this investigation, our aim is to assess the safety, feasibility, and advantages of the procedure, which may evolve to be as common as the conventional LRP after continued adaptations. To our knowledge, we are the first to report this novel “three-port” trocar placement technique for LRP.

## Methods

Between November 2010 and June 2015, we retrospectively reviewed the records of PCa patients at our institution. A total of 300 patients receiving the surgical treatment (three-port LRP, the conventional four-five-port LRP, ORP, and RARP) were selected. Patients who had undergone previous major abdominal surgery, metastatic disease, or radiation therapy were excluded from the trial. These patients were divided into 4 groups based on the detailed surgical approach applied. Group A (three-port LRP) consisted of 144 patients (48.0%), group B (conventional four-five-port LRP) consisted of 88 patients (29.3%), group C (ORP) consisted of 57 patients (19.0%), and group D (RARP) consisted of 11 patients (3.7%).

All of the groups were compared according to perioperative parameters, such as age, BMI, prostate volume, prostate serum antigen level, Gleason score, OT, EBL, drainage days, hospitalization days, surgical complications, postoperatively pathological stages, and PSM. The OT was calculated from skin incision to the skin closure. The intraoperative EBL was calculated by anesthesiologists. The complications were recorded according to the Clavien-Dindo grading system. The postoperative pathological tumor stage was established according to TNM 2018. To evaluate the learning speed of the three-port LRP, a learning curve was also analyzed by dividing these 144 consecutive patients into the early stage (72 patients) and late stage (72 patients) according to their surgical periods.

All statistical tests were carried out with the program SPSS v16.0. For statistical analysis, categorical variables were summarized as the frequency and percent. Continuous variables were summarized as the mean ± standard deviation for normally distributed data. The groups were compared for continuous variables using the independent *t* test and for proportions using the Pearson chi-square test or Fisher’s exact test. The statistical significance level for each hypothesis was established at 0.05.

**Fig. 1:**
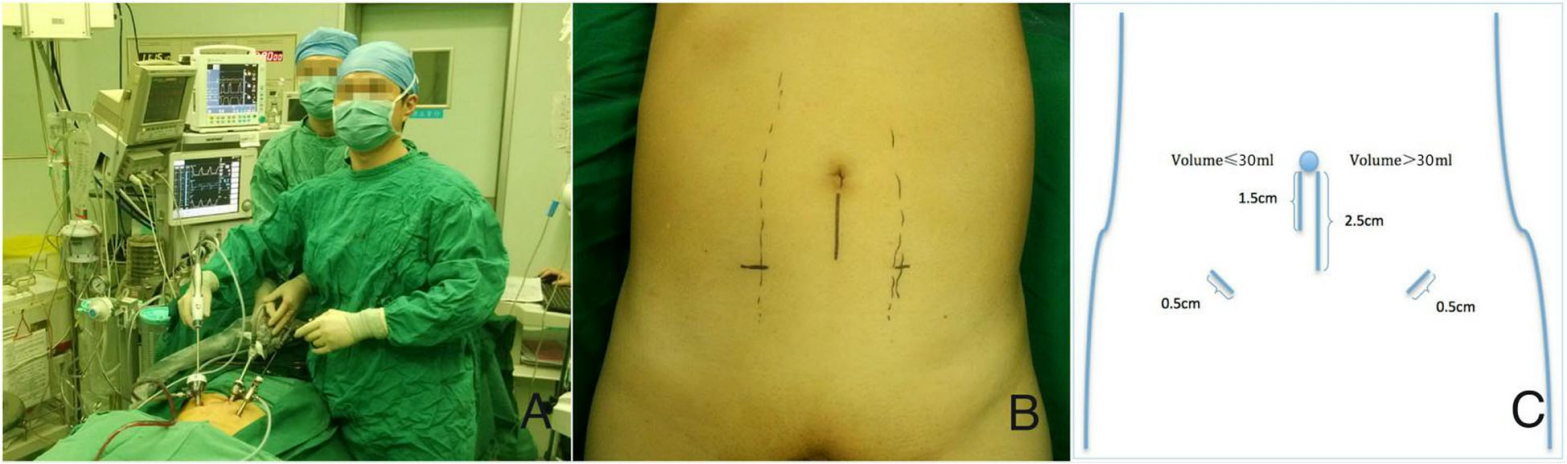
Images of the external cavity. **a** With an assistant holding the laparoscope, the surgeon alone completed all procedures of the operation. **b** Only three trocars were placed, including one just in the umbilical region and two on the left/right lateral margin of the rectus abdominis muscle with a length of 2 finger-breadths below the umbilicus. **c** The total lengths of incisions were 1.5 + 0.5 + 0.5 = 2.5 cm for the prostate volume (evaluated by preoperative B-ultrasonography) ≤ 30 ml or 2.5 + 0.5 + 0.5 = 3.5 cm for the prostate volume > 30 ml

All of the patients in group A were performed by an extraperitoneal approach with only three trocars involved by subtracting an additional incision at the McBurney point. A sub-umbilical incision (1.5 cm for the prostate volume ≤ 30 ml or 2.5 cm for the prostate volume > 30 ml) was initially made through the skin, subcutaneous tissue, and rectoabdominal fascia. Afterwards, a 10-mm trocar was introduced gently through this incision, and the extraperitoneal space was then extended carefully up to the pelvis. Under the direct optic vision through this trocar, the other two incisions for trocar placement were performed successively: one on the right and one on the left lateral margin of the rectus abdominis muscle with a length of 2 finger-breadths below the umbilicus. The operation was performed by only two surgeons (Fig. 1a). The detailed trocar locations are revealed in Fig. 1b, c.

## Results

The detailed patient demographics and outcome characteristics are listed in Table 1. The groups were comparable with regard to all of the preoperative characteristics except for the relatively smaller prostate volume in group A. The three-port LRP operations were performed successfully with only 8 cases of conversion to the conventional LRP. None of any severe complications or conversion to ORP occurred. Only five mild complications of postoperative ileus and anastomosis leak occurred, but the patient recovered with conservative methods. In group A, the mean OT was 113.8 min, the mean EBL was 94.2 ml, the mean drainage day duration was 4.0 days, the mean postoperative hospital stay was 5.1 days, and 27.8% of the PSMs were positive. The differences of OT, EBL, drainage days, hospitalization, and transfusion in group A were statistically significant among the majority of the other groups (*p* < 0.05). However, the other parameters including postoperative complications, postoperative pathological stages, Gleason scores, and PSMs were not significant (*p* > 0.05) among different groups. Above all, group A was associated with a shorter OT, less EBL and blood transfusion, and fewer drainage days and hospitalization.

**Table 1:** Patient demographics and outcome characteristics of patients among groups

Variables	Group A	Group B	Group C	Group D	*p* value	*p* value after comparison		
						A vs B	A vs C	A vs D
No. patients	144	88	57	11	–	–	–	–
Age (years)	66.0 ± 7.1	67.7 ± 6.8	66.5 ± 6.3	68.6 ± 8.5	0.244	–	–	–
BMI (kg/m^2^)	24.2 ± 2.6	24.8 ± 2.9	24.6 ± 2.7	23.6 ± 2.9	0.253	–	–	–
Smoking history (%)	16 (11.1%)	15 (17.0%)	6 (10.7%)	2 (18.2%)	0.470	–	–	–
ADT history (%)	16 (11.1%)	5 (5.7%)	1 (1.8%)	1 (9.1%)	0.100	–	–	–
PSA level (ng/ml)	13.4 ± 12.1	15.2 ± 12.6	14.1 ± 27.3	15.2 ± 17.7	0.867	–	–	–
Prostate volume (ml)	35.2 ± 16.0	42.6 ± 26.7	46.7 ± 25.2	36.6 ± 11.5	0.003	0.118	0.013	0.999
Preoperative Gleason scores by puncture (%)								
< 7	37 (25.7%)	30 (34.1%)	20 (35.1%)	5 (45.4%)	0.113	–	–	–
= 7	88 (61.1%)	38 (43.2%)	28 (49.1%)	4 (36.4%)				
> 7	19 (13.2%)	20 (22.7%)	9 (15.8%)	2 (18.2%)				
EBL (ml)	94.2 ± 73.4	216.7 ± 173.2	1247.9 ± 1137.2	150.0 ± 130.4	< 0.001	< 0.001	< 0.001	0.661
OT (min)	113.8 ± 21.1	130.6 ± 30.3	240.1 ± 52.1	214.4 ± 38.8	< 0.001	0.002	< 0.001	< 0.001
Drainage days (days)	4.0 ± 2.6	3.7 ± 2.9	6.4 ± 5.7	4.5 ± 1.6	< 0.001	0.973	0.018	0.913
Hospitalization days (days)	5.1 ± 2.9	5.2 ± 3.4	8.8 ± 5.8	6.3 ± 2.5	< 0.001	1.000	< 0.001	0.647
Transfusion (ml)	0	4.5 ± 42.6	652.6 ± 789.8	0	< 0.001	0.897	< 0.001	–
Complications								
0	130 (96.3%)	81 (92.0%)	52 (91.2%)	10 (90.9%)	0.104	–	–	–
I	4 (3.0%)	5 (5.7%)	3 (5.3%)	0				
II	1 (0.7%)	0	2 (3.5%)	1 (9.1%)				
III	0	2 (2.3%)	0	0				
Postoperatively pathological stages (%)								
T2	87 (60.4%)	41 (46.6%)	34 (59.6%)	5 (45.5%)	0.161	–	–	–
T3	57 (39.6%)	47 (53.4%)	23 (40.4%)	6 (54.5%)				
PSM (%)	40 (27.8%)	27 (30.7%)	19 (33.3%)	4 (36.4%)	0.792	–	–	–
Postoperative Gleason scores by operation (%)								
< 7	10 (6.9%)	9 (10.2%)	8 (14.0%)	1 (9.1%)	0.242	–	–	–
= 7	112 (77.8%)	56 (63.6%)	39 (68.4%)	8 (72.7%)				
> 7	22 (15.3%)	23 (26.1%)	10 (17.6%)	2 (18.2%)				

Group A, three-port LRP; group B, conventional four-five-port LRP; group C, ORP; group D, RARP

*No.* number, *BMI* body mass index, *PSA* prostate-specific antigen, *EBL* estimated blood loss, *OT* operative time, *PSM* positive surgical margin

**Fig. 2:**
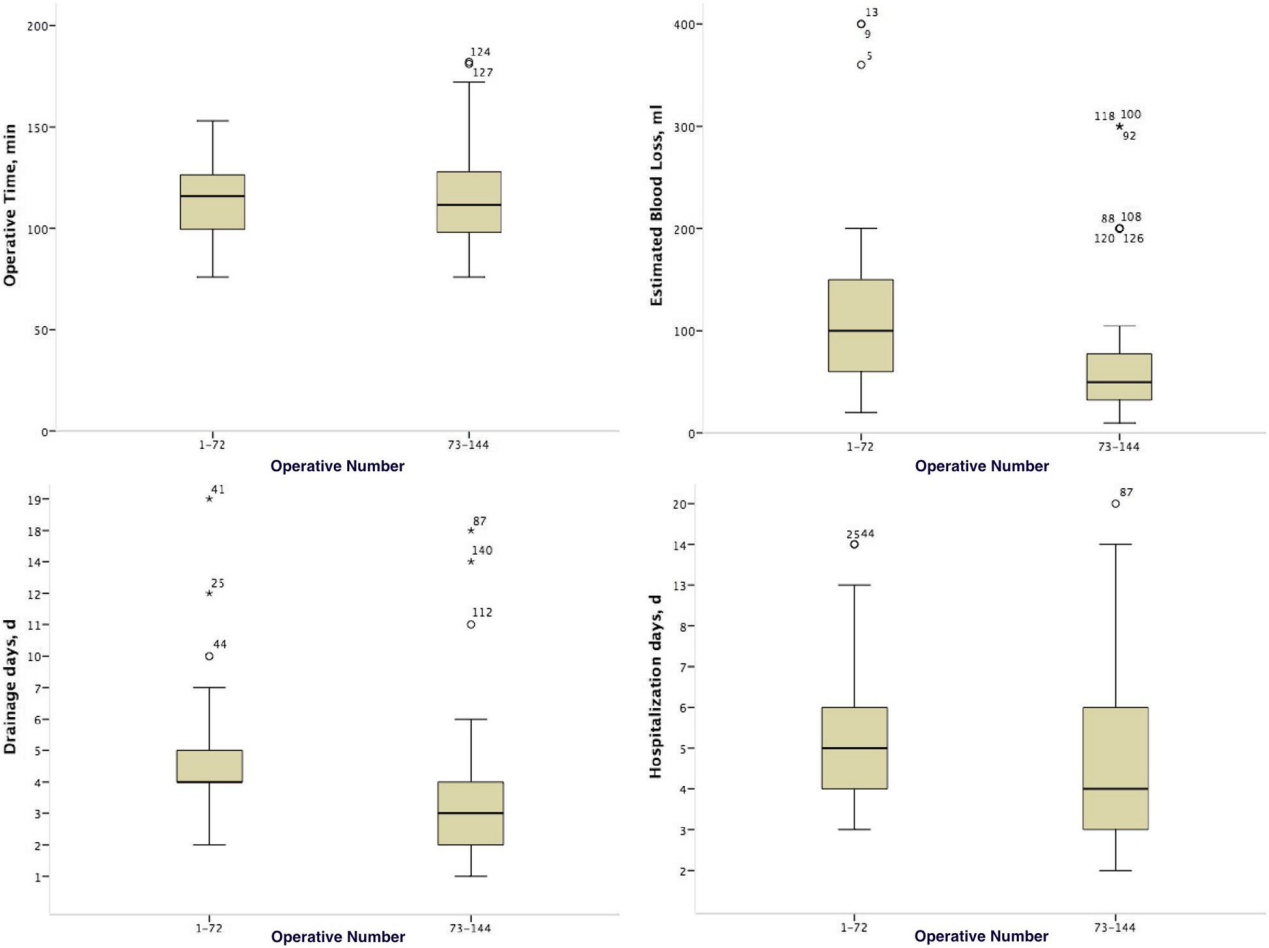
A comparison of three-port LRP between the early stage (initial 72 cases) and the late stage (next 72 cases) for the parameters of OT, EBL, drainage days, and hospitalization days

After undergoing the early stage of a learning curve analysis in three-port LRP, an improvement in the OT, EBL, drainage days, and hospitalization is reflected in Fig. 2. Among them, the EBL was evidently decreased for the initial 72 cases than for the next 72 cases. Although the OT, drainage days, and hospitalization of the initial 72 cases were not significantly different from those of the next 72 cases, a tendency towards more superior outcomes was still observed in the late stage.

## Discussion

Because of the familiar route and reliable touch [12], ORP has always been the “gold standard” treatment for PCa [13], but since 1991, LRP for PCa treatment has been widely disseminated in an attempt to decrease morbidity compared with the traditional ORP [14], even though with high-risk PCa patients [15]. With the improvement of the modern technology and the advent of robotic instruments, RARP was promptly applied in the USA and some developed European countries as the most common extirpative treatment for PCa [16].

Nevertheless, the increased technical effort with a longer robot docking time and the increased cost associated with the robot-assisted operation cannot be ignored. It has been demonstrated that over 10 years, RARP was on average more costly than LRP and ORP [17]. Especially in the developing countries such as China, the healthcare resources are in heavy shortage and medical insurance fails to cover the fees on robot-assisted operations. The high cost has also led a number of authorities to question the value of RARP to patients and health care systems. Unfortunately, this unpleasant situation cannot be improved by the surgeons or the hospital itself, but by the economists and politicians. Therefore, the RARP may not be generalizable to the developing countries and community settings. In developing countries such as China, choosing LRP instead of RARP remains common due to the robotic medical expenses that the national health insurance system does not cover. Factually, the standard laparoscopic technique still continues to be practiced in a number of centers due to the higher total hospitalization costs of RARP [18, 19].

As for the extensive application of LRP and RARP, it can be further divided into the conventional four-five port and a more minimally invasive single port. Single-port technique is associated with reductions in the number of transcutaneous access points, reducing incision-related complications and improving cosmesis [20], with single-port RARP initially reported in 2019 [21, 22]. However, due to a loss of triangulation, small operative space and instrument clashes with some doubtful factors on the safety of the procedure and the extended OT, concentrating the incisions at a single site limits the range of motion and makes visualization difficult, which is a huge challenge even for an experienced surgeon. Additionally, the increased cost related to the use of disposable single-port elements must also be taken into account when considering the application of this technique [23].

Du et al. [24] insisted that RARP is more beneficial for PCa patients than LRP and ORP by a system review and meta-analysis. Nevertheless, the extended OT and financial burden of RARP must not be easily neglected. As is well known, prolonged OT is associated with an increased risk of complications in PCa patients [25]. To overcome the above limitations and a loss of triangulation without efficient cooperation by three unfamiliar surgeons of the conventional four-five-port LRP, our team modified the conventional LRP technique and now performs three-port LRP as our first-line treatment for PCa. In our views, three-port LRP combines the advantages of lower cost, faster OT, lower complication rates, and acceptable incision cosmesis. Using laparoscopic vision, the surgeon can detect certain features that cannot be realized accurately and vividly by the RARP. Furthermore, the three-port equilateral triangle can avoid a narrow space, which is a remarkable disadvantage when executing a single-port technique. Above all, three-port LRP is the best combination of direct contact with the surgeon’s observations, a spacious cavity and efficient coordination in clinical practice. In three-port LRP, the concept of triangulation implies an instrument positioning schema that provides an optimal relationship between the camera and the working instrument. With the bipolar instrument and the laparoscopic traction forceps both in the surgeon’s hand, this setup can promote accurate retraction and rapid hemostasis. Some important procedures can be achieved promptly and efficiently only by the surgeon himself. In our clinical practice, three-port LRP is achieved by an extraperitoneal approach instead of a transperitoneal route in view of its lower rate of postoperative complications. Wang et al. [26] and Ragavan et al. [27] have also supported its advantage of extraperitoneal manipulations based on a meta-analysis of LRP and RARP, respectively.

In Table 2, combining our three-port LRP data with other urologists’ experience, it can be clearly revealed that our mean OT is significantly shorter. Likewise, the parameters of EBL and the rates of surgical complications are also superior to the other urologists. The perioperative data in our investigation appeared more excellent, which can be explained by three points: (1) a more quick recovery time with less trocar placement and incisions, (2) the risks of faulty operation can be decreased evidently due to the inflexible and excessive traction by an inexperienced and unskilled assistant, and (3) the triangle operation in accordance with the human engineering principle makes the surgeon feel more comfortable and correspondingly reduce the fatigue.

**Table 2:** A synopsis of published series on the surgical treatment of PCa

Reference	Treatment	No. of patients	OT (min)	EBL (ml)	Drainage days (days)	Hospitalization days (days)	Complications (%)	PSM (%)
Zhu et al. [28]	Single-port LRP	6	252.5	300	11	NA	33	0
Zhang et al. [29]	Two-port LRP	15	170.1	100.7	5.7	NA	13.3	13.3
İnkaya et al. [30]	ORP	128	160	1600	NA	9	81.25	33.04
Yaxley et al. [15]	ORP	151	234.34	1338.14	8.42	3.27	10.6	8.0
İnkaya et al. [30]	Conventional LRP	48	248	183	11.6	3.68	8.3	12.5
Papachristos et al. [31]	Conventional LRP	100	195	300	NA	2	12	13
Sirisopana et al. [32]	Conventional LRP	241	210	500	NA	6	29.05	40.63
Johnson et al. [33]	Conventional LRP	544	213	NA	10.6	3.2	19.1	27.6
Qi et al. [34]	Conventional LRP	74	143.8	316.89	4.77	7.09	NA	45.9
Yaxley et al. [15]	RARP	157	222.03	443.74	8.21	1.55	4.5	11.0
İnkaya et al. [30]	RARP	778	206	172	9.2	3.02	2.4	17.0
Papachristos et al. [31]	RARP	100	195	300	NA	2	9	10
Sirisopana et al. [32]	RARP	295	200	300	NA	6	8.81	39.15
Johnson et al. [33]	RARP	1081	135	NA	13.3	2.9	16.4	22.5
Tasci et al. [35]	RARP	1499	181.9	225.4	2.3	2.9	6.1	14.1
Kaouk et al. [36]	Single-port RARP	10	197.5	143	8	0	0	50.0
Dobbs et al. [37]	Single-port RARP	10	234	65	1.3	1.3	0	20.0
Our series	Three-port LRP	144	113.8	94.2	4.0	5.1	3.7	27.8

*PCa* prostate cancer, *ORP* open radical prostatectomy, *LRP* laparoscopic radical prostatectomy, *RARP* robotic-assisted radical prostatectomy, *No.* number, *OT* operative time, *EBL* estimated blood loss, *PSM* prostate surgical margin, *NA* not available

Admittedly, our study has several limitations. This study is retrospective and non-randomized, which clearly biases subsequent analysis. Though the short-term results are encouraging in three-port LRP, the oncological and functional outcomes in the long-term follow-up are still not clear. A series of scientists [38–40] have proposed that there is no evidence to inform the comparative effectiveness of LRP or RARP compared with ORP for oncologic and functional outcomes, which might indicate that three-port LRP can still guarantee an ideal oncologic control balanced with an excellent cosmesis. Hence, further study is still necessary to validate and extrapolate this application.

## Conclusions

By making a comparison between three-port LRP and other surgical techniques, due to its less OT, EBL, drainage days, hospitalization, and transfusion with a shorter learning curve, the novel “three-port” LRP should be recommended and popularized in the clinical practice.

## Data Availability

N/A

## Abbreviations

LRP: Laparoscopic radical prostatectomy
PCa: Prostate cancer
ORP: Open RP
RARP: Robotic-assisted RP
OT: Operative time
EBL: Estimated blood loss
PSM: Prostate specimen margins
BMI: Body mass index
